# Ubiquitin-specific protease USP2-45 acts as a molecular switch to promote α_2_δ-1-induced downregulation of Ca_v_1.2 channels

**DOI:** 10.1007/s00424-014-1636-6

**Published:** 2014-11-05

**Authors:** Jean-Sebastien Rougier, Maxime Albesa, Ninda Syam, Guillaume Halet, Hugues Abriel, Patricia Viard

**Affiliations:** 1 Department of Neurosciences, Physiology, and Pharmacology, University College London, London, WC1E 6BT UK; 2 Department of Clinical Research, University of Bern, Bern, 3010 Switzerland; 3 CNRS, UMR 6290, Institut de Génétique et Développement de Rennes, 35043 Rennes, France; 4 Université Rennes 1, UEB, IFR 140, Faculté de Médecine, 35043 Rennes, France

**Keywords:** Calcium channels, Ubiquitin-specific proteases 2-45, Ubiquitylation, α_2_δ-1 Subunit

## Abstract

Availability of voltage-gated calcium channels (Ca_v_) at the plasma membrane is paramount to maintaining the calcium homeostasis of the cell. It is proposed that the ubiquitylation/de-ubiquitylation balance regulates the density of ion channels at the cell surface. Voltage-gated calcium channels Ca_v_1.2 have been found to be ubiquitylated under basal conditions both in vitro and in vivo. In a previous study, we have shown that Ca_v_1.2 channels are ubiquitylated by neuronal precursor cell-expressed developmentally downregulated 4 (Nedd4-1) ubiquitin ligases, but the identity of the counterpart de-ubiquitylating enzyme remained to be elucidated. Regarding sodium and potassium channels, it has been reported that the action of the related isoform Nedd4-2 is counteracted by the ubiquitin-specific protease (USP) 2-45. In this study, we show that USP 2-45 also de-ubiquitylates Ca_v_ channels. We co-expressed USPs and Ca_v_1.2 channels together with the accessory subunits β_2_ and α_2_δ-1, in tsA-201 and HEK-293 mammalian cell lines. Using whole-cell current recordings and surface biotinylation assays, we show that USP2-45 specifically decreases both the amplitude of Ca_v_ currents and the amount of Ca_v_1.2 subunits inserted at the plasma membrane. Importantly, co-expression of the α_2_δ-1 accessory subunit is necessary to support the effect of USP2-45. We further show that USP2-45 promotes the de-ubiquitylation of both Ca_v_1.2 and α_2_δ-1 subunits. Remarkably, α_2_δ-1, but not Ca_v_1.2 nor β_2_, co-precipitated with USP2-45. These results suggest that USP2-45 binding to α_2_δ-1 promotes the de-ubiquitylation of both Ca_v_1.2 and α_2_δ-1 subunits, in order to regulate the expression of Ca_v_1.2 channels at the plasma membrane.

## Introduction

Ubiquitylation is a post-translational modification which has been shown to regulate the availability of both intracellular and membrane proteins [1, 35]. Ubiquitin, a small protein of 8 kDa, is attached by ubiquitin ligases onto lysine residues of target proteins. Equally important is the reverse process operated by de-ubiquitylases (DUBs) [29, 32]. The ubiquitylation and therefore the fate of the targeted proteins result from the balanced action of both families of enzymes. A multitude of ubiquitin ligases and de-ubiquitylases have already been identified, and one current challenge is to identify their associated targets. The human genome encodes for more than 100 de-ubiquitylases [29, 32]. They have been classified into five families according to the active sites used to perform the de-ubiquitylation process: cysteine or zinc active sites. The zinc active site is found in JAB1/MPN/MOV34 metalloenzymes, and the cysteine active site is found in ubiquitin C-terminal hydrolase, ovarian tumour proteases, Machado-Josephin domains and ubiquitin-specific proteases (USPs) [29, 32]. Interestingly, USP2-45 had been shown to de-ubiquitylate the epithelial sodium channel (ENaC) [38] and regulate its expression at the plasma membrane. The same research group further reported that USP2-45 physically interacts with the neuronal precursor cell-expressed developmentally downregulated 4 (Nedd4-2) ubiquitin ligases [30] known to ubiquitylate ENaC [24, 44]. Moreover, both USP2-45 and USP2-69 counteracted the Nedd4-2-mediated ubiquitylation of cardiac potassium channels [26]. We have previously shown that another closely related isoform, Nedd4-1, ubiquitylates Ca_v_1.2 channels [36]. In spite of Nedd4-1 and Nedd4-2 having selective targets [24, 36], these studies suggest that there may be a reciprocity between the two families of Nedd4 ubiquitin ligases and USP de-ubiquitylases, which lead us to firstly investigate USP2-45 as a potential regulator of Ca_v_1.2 channels. USP2-45 is one of the two isoforms encoded by the USP2 gene which is alternatively spliced to give USP2-45 (45 kDa) and USP2-69 (69.5 kDa) [29]. Both splice variants share an identical catalytic core, but USP2-69 has a longer N-terminal domain which may possibly regulate USP2-69 subcellular localization or act as an auto-inhibitory domain [30]. Gousseva and Baker have shown that USP2-45 and USP2-69 messenger RNAs (mRNAs) are expressed together in mouse testis, skeletal muscle and heart [22]. USP2-45 mRNA is also present in the brain, liver and kidney [22]. Increased expression of USP2 was reported in prostate tumours [31]. Tissue expression of USP2 can also be modulated by signalling pathways, as shown by Fakitsas et al. [15] who demonstrated that aldosterone induces the expression of USP2-45 in the cortical collecting duct of the kidney.

Our study investigates whether USP2 regulates voltage-gated calcium Ca_v_1.2 channels. These widely expressed channels constitute the main pathway for calcium entry into vascular and cardiac myocytes and are a major target for the treatment of cardiovascular diseases [27, 40]. Ca_v_1.2 channels also contribute to neuron excitability and are involved in the control of gene transcription [13]. In addition to the main pore-forming Ca_v_1.2 subunit, Ca_v_1.2 channels also contain accessory subunits: β and α_2_δ, which regulate both the gating properties and trafficking of the channels [9, 41, 17, 7, 6]. Ca_v_1.2 channels are predominantly associated with β_2_ [10, 43] and α_2_δ-1 [5, 28] in the heart. We, and others, have previously shown that these three Ca_v_1.2 subunits are ubiquitylated in vitro and in vivo [25, 36]. Here, we found that both USP2-45 and USP2-69 regulate cloned cardiac Ca_v_1.2 channels expressed in mammalian cell lines. The main difference between the two USP2 isoforms resides in their N-terminal domain which was suggested to direct target specificity [30] as reported for ENaC channels which are not sensitive to USP2-69 [15]. The similar reduction of Ca_v_ currents obtained with either splice variant suggests that Ca_v_ channels are the target of both isoforms and that the minimum sequence required for Ca_v_ current reduction resides within the short variant form of USP. Hence, we focussed our investigation on USP2-45-induced regulation of Ca_v_ channels. Our study reveals a new role for α_2_δ-1 subunits in binding USP2-45 and promoting the de-ubiquitylation of both α1 and α_2_δ-1 subunits. USP2-45-induced Ca_v_ regulation leads to a decrease of Ca_v_1.2 channels available at the plasma membrane.

## Methods

### DNA constructs

Rabbit Ca_v_1.2 (cardiac isoform α1c) (P15381.1), β_2b_ (P54288) and α_2_δ-1a (P13806) complementary DNAs (cDNAs) subcloned into pCARDHE, pBH17 and pCA1S, respectively, were gifts from Dr. G.S. Pitt (Department of Medicine, Division of Cardiology, Duke University Medical Center, Durham, NC, USA). Mouse USP2-45 (NM 198091) with and without S-tag and the mutant USP2-45C67A cDNAs subcloned into pcDNA_3.1_ were gifts from Prof. O. Staub (Department of Pharmacology and Toxicology, Lausanne, Switzerland).

### Transfections

For electrophysiological studies, T25-cm^2^ flasks of tsA-201 cells were transiently co-transfected using Fugene® 6 mix reagent (Roche Diagnostics, IN, USA) with 0.3 μg of each subunit of voltage-gated calcium channel (Ca_v_1.2, β_2_ and α_2_δ-1 subunits; ratio 1:1:1) and 1.0 μg of other constructs or empty vector. An equivalent amount of pcDNA3.1 DNA was added to the transfection mix to compensate for the absence of DNA encoding Ca_v_ subunits or USPs when omitted. All transfections included 0.5 μg of cDNA encoding CD8 antigen as a reporter gene. Anti-CD8 beads (Dynal®, Oslo, Norway) were used to identify transfected cells, and only decorated cells were analysed. For biochemistry experiments, T75-cm^2^ flasks of HEK-293 cells were transfected using Lipofectamine LTX® (Invitrogen, Basel, Switzerland) according to the manufacturer’s instructions. The ratio of cDNAs/Lipofectamine was 10 μg cDNAs/30 μl Lipofectamine. The ratio of the different constructs was similar to those used in patch clamp experiments. In biochemistry experiments, an additional control was performed by using the empty vector pcDNA3.1 only in Ca_v_-untransfected cells. Cells were used 48 h after transfection.

### Electrophysiology

Whole-cell currents were measured at room temperature (22–23 °C) using an Axopatch200B amplifier (Axon Instruments, Union City, CA, USA). TsA-201 cells were replated 24 h post-transfection onto 35-mm plastic Petri dishes and recorded from 24 h post-replating. The internal pipette solution was composed of (in mM) 60 CsCl, 70 cesium aspartate, 1 MgCl_2_, 10 HEPES, 11 EGTA and 5 Mg-ATP, pH 7.2 with CsOH. The external solution contained (in mM) 130 NaCl, 5.6 KCl, 5 to 20 BaCl_2_, 1 MgCl_2_, 10 HEPES and 11 d-glucose, pH 7.4 with NaOH. The osmolarity, assessed using a Löser micro-osmometer (Giessen, Marburger, Germany), was 295 mOsm l^−1^ for both the internal solution and the 5 mM BaCl_2_ external solution and 325 mOsm l^−1^ for the 20 mM BaCl_2_ external solution. In experiments performed without β subunits which are known to promote Ca_v_ targeting, we used the higher 20 mM BaCl_2_ solution to compensate for the lack of β subunits which greatly reduced Ca_v_ currents. 5 mM BaCl_2_ was used for all other experiments. Data were analysed using pClamp software, version 9.2 (Axon Instruments, Union City, CA, USA), and Origin software, version 7.5 (OriginLab® corporation, Northampton, MD, USA). Barium current densities (pA/pF) were calculated by dividing the peak current by the cell capacitance. I-V relationship (IV) were fitted with the following equation *I* = (g(*V*h − *V*_rev_)) / (1 + exp[(*V*h − *V*_50_) / *k*]), in which *I* is the normalized peak current density (pA/pF) at a given holding potential (*V*h), *V*_50,act_ is the voltage at which half of the channels are activated, *k* is the slope factor, *V*_rev_ is the reversal potential and *g* is the conductance. The maximal conductance *G*_max_ was calculated from the maximal peak current density.

### Western blots

T75-cm^2^ flasks of HEK-293 cells were lysed in 1.0 ml of lysis buffer (50 mM HEPES pH 7.4, 150 mM NaCl, 10 % glycerol, 1 % triton, 1 mM EGTA supplemented with 10 mM *N*-ethylmaleimide and protease inhibitors). Protein concentration was systematically determined by performing a Bradford assay (Coo protein dosage kit; Interchim, Montluçon, France). Eighty micrograms of proteins were loaded on an SDS-PAGE gel. Protein transfer was done with the dry system transfer iBlot® from Invitrogen (Invitrogen, Basel, Switzerland). Immunoblotting was accomplished by using the SNAP id® system of Millipore (Millipore, Zug, Switzerland). Fluorescent secondary antibodies were used, and detection was realized using the LICOR system® (Lincoln, USA). The intensity of the bands was quantified with the Odyssey software (LICOR).

### Surface biotinylation assay

HEK-293 cells were washed twice with PBS 1×, 48 h after transfection, and then treated for 30 min at 4 °C with 4 ml non-permeant biotin per T75-cm^2^ flasks (1 mg/ml; EZ link Sulfo-NHS-SS-Biotin; Pierce, Rockford, USA). Biotin binds to the lysine residues of proteins exposed to the extracellular medium. Cells were washed three times with cold PBS 1× containing 0.2 M glycine and lysed with 1 ml/dish of lysis buffer. The cells were solubilized for 1 h on a wheel at 4 °C and centrifuged for 30 min at 20,000*g* at 4 °C. Supernatants were recovered, and protein concentrations were quantified by the Bradford method. One hundred micrograms of the lysates were used to assess the transfection efficiency. Proteins inserted at the plasma membrane were then selectively pulled down with 50 μl of streptavidin sepharose beads (GE Healthcare Europe, Glattbrugg, Switzerland) added to 1 mg of total proteins before incubation for 2 h on a wheel at 4 °C. The beads were washed five times with lysis buffer, and beads were resuspended in 2.5× sample buffer (Invitrogen, Basel, Switzerland). Eluted proteins were analysed by Western blot.

### Immunoprecipitation

Transiently transfected HEK-293 cells in P100 plates were harvested after 48-h incubation and lysed with 1× cold Ubi lysis buffer (50 mM HEPES pH 7.4, 150 mM NaCl, 1 mM EGTA, pH 8.0, 10 % glycerol, 1× EDTA-free complete protease inhibitor cocktail (Roche, Mannheim, Germany); 2 mM *N*-ethylmaleimide/NEM (Sigma-Aldrich, St. Louis, MO, USA); 10 mM iodoacetamide/IAA (Sigma-Aldrich, St. Louis, MO, USA)) containing 1 % Triton X-100 for 1 h at 4 °C. Cell lysates were then centrifuged at 16,000*g* at 4 °C for 15 min. Two milligrams of the supernatant (lysate) was incubated at 4 °C for 24 h with anti-Ca_v_1.2 channel subunits antibodies. One volume of 1× cold Ubi lysis buffer without Triton X-100 (to obtain a final concentration of 0.5 % Triton X-100) was also added in the mix. On the next day, the lysate-antibody mix was transferred to a microcentrifuge tube containing 50 μg (1:1 beads to lysis buffer ratio) of Protein G Sepharose beads (GE Healthcare, Uppsala, Sweden) which were previously washed three times with 1× cold Ubi lysis buffer containing 0.5 % Triton X-100. After adding fresh 1× EDTA-free complete protease inhibitor cocktail, the mix was incubated overnight at 4 °C. The beads were subsequently washed five times (4 °C; 3,000 rpm) with 1× cold Ubi buffer containing 0.3 % Triton X-100 before elution with 50 μl of 2× NuPAGE sample buffer with 100 mM DTT at 37 °C for 30 min. These samples are designated as immunoprecipitation (IP) fractions. The input fractions were resuspended with 4× NuPAGE sample buffer with 100 mM DTT to give a concentration of 1 mg/ml and incubated at 37 °C for 30 min. All lysis and incubation steps, except elution in sample buffer, were performed in the dark.

### Pull down of ubiquitylated proteins

Expression of GST-S5a fusion proteins in *Escherichia coli* bacteria was induced with 0.2 mM isopropyl β-d-1-thiogalactopyranoside for 4 h at 29 °C. Cells were harvested by centrifugation and resuspended in lysis buffer (200 mM Tris pH 7.5, 250 mM NaCl, 1 mM EDTA, 0.5 % Igepal). Supernatant from 15 min of centrifugation at 13,000*g* (4 °C) was incubated 1 h in the presence of GSH-sepharose beads at 4 °C. Beads were then washed three times with lysis buffer and used in pull-down experiments. One milligram of total protein (HEK-293 lysates) was added to 50 μg of GST-S5A beads and incubated for 2 h at 4 °C. After washing the beads three times with lysis buffer, precipitated proteins were eluted with sampling buffer (Invitrogen, Basel, Switzerland) and analysed by Western blot.

### Pull down of S-tagged USP2-45

One milligram of HEK-293 cells lysate was incubated for 1 h at 4 °C with 1 μl of biotinylated S-protein (Merck biosciences, Darmstadt, Germany), followed by 1-h incubation at 4 °C with streptavidin sepharose beads (GE Healthcare Europe, Glattbrugg, Switzerland). The beads were washed five times with lysis buffer, and beads were resuspended in 2.5× sample buffer (Invitrogen, Basel, Switzerland). Eluted proteins were analysed by Western blot.

### Antibodies

Antibody against Ca_v_1.2 (ACC003; Alomone, Jerusalem, Israel) was used at a dilution of 1/200. Antibody against β_2b_ (ab54920; Abcam, Cambridge, UK) was used at a dilution of 1/200. Antibody against α_2_δ-1 (ab2864; Abcam, Cambridge, UK) was used at a dilution of 1/1,000. FK2 antibody was used at the dilution of 1/500 (PW8810, Enzo Life Sciences, Lausanne, Switzerland). USP2 antibody used at a dilution of 1/500 was a gift from Prof. O. Staub (Department of Pharmacology and Toxicology, Lausanne, Switzerland). Monoclonal antibody raised against actin was purchased from Sigma-Aldrich (Sigma-Aldrich Chemie, Postfach, Switzerland) and used at a dilution of 1/1,000. Antibody raised against S-tag was purchased from Abcam (ab18588; Abcam, Cambridge, UK) and used at a dilution of 1/200.

### Statistical analysis

Two-tailed Student’s *t* test was used to compare two groups of data. One-way ANOVA was used to compare three or more groups. Data are represented as mean ± SEM. *P* < 0.05 was considered significant.

## Results

### Ca_v_1.2 currents are downregulated by USP2-45

**Fig. 1:**
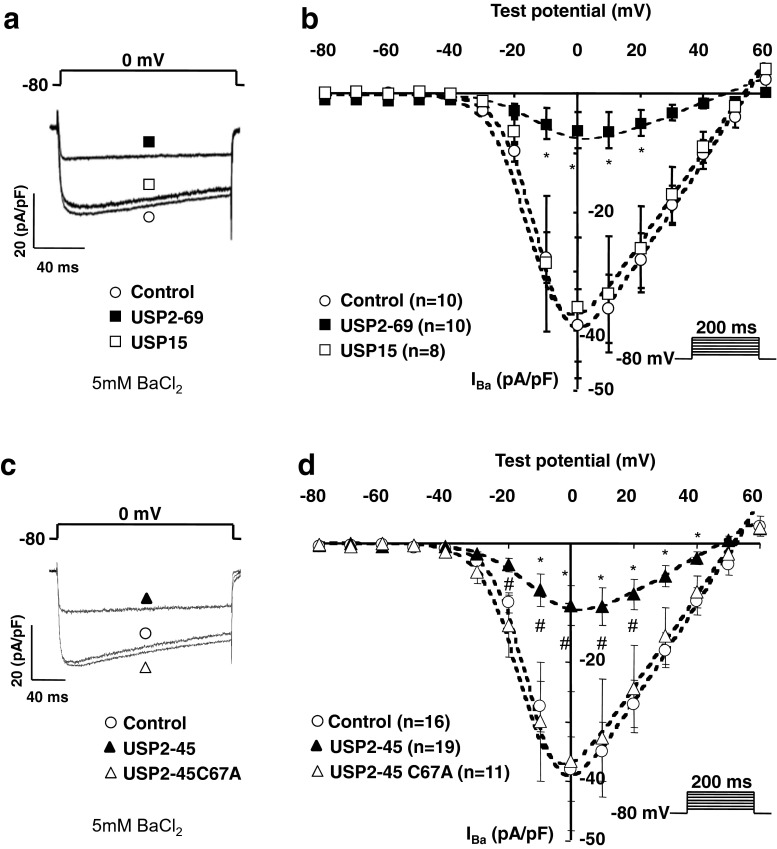
Effect of USP de-ubiquitylases on Ca_v_1.2 currents. **a** Representative whole-cell current traces and **b** corresponding current-voltage (I-V) relationships in Ca_v_1.2/β_2_/α2δ-1 channels transfected alone with control (*white circle* (control)), or with USP2-69 (*black square*) or USP15 (*white square*) in tsA-201 cells. The maximal conductance and reversal potential were calculated with the I-V fit described in ‘Methods’. *G*_max_ was specifically decreased in USP2-69 (0.3 ± 0.2 nS/pF versus 0.8 ± 0.1 nS/pF in control; *P* < 0.05), but not in USP15-transfected cells (0.8 ± 0.2 nS/pF; NS versus control), whilst *V*_rev_ was not modified by either USP2-69 (48 ± 3 mV versus 54 ± 3 mV in control; NS) or USP15 (46 ± 3 mV; NS versus control). The values for *V*_50,act_ and slope factors are indicated in the results section. One-way ANOVA statistical analysis compared control with USP2-69 or USP15 (*p < 0.05). The number of cells is indicated in parentheses. **a** Representative whole-cell current traces and **b** corresponding current-voltage relationships in tsA-201 cells transfected with Ca_v_1.2/β_2_/α2δ-1 channels alone (*white circle* (control)), or with USP2-45 (*black triangle*) or the catalytically inactive mutant USP2-45 C67A (*white triangle*). *G*_max_ was decreased by USP2-45 (0.4 ± 0.1 nS/pF versus 0.8 ± 0.1 nS/pF in control; *P* < 0.05), but not by USP2-45 C67A (0.8 ± 0.2 nS/pF; NS versus control), whilst *V*_rev_ was not modified by either USP2-45 (44 ± 4 mV versus 54 ± 3 mV in control; NS) or USP2-45 C67A (52 ± 1 mV; NS versus control). The values for *V*_50,act_ and slope factors are indicated in the results section. One-way ANOVA statistical analysis compared control with USP2-45 (*p < 0.05) and USP2-45 C67A with USP2-45 (#*p* < 0.05). In all experiments, 5 mM BaCl_2_ was used to record Ca_v_ currents

We expressed Ca_v_1.2 together with the accessory subunits β_2_ and α_2_δ-1 in tsA-201 cells and assessed the effect of the two USP2 variants and USP15 on Ca_v_ currents. Figure 1 shows that co-expressing either USP2-69 (Fig. 1a, b) or USP2-45 (Fig. 1c, d) reduced whole-cell Ca_v_ current densities (respectively by 83 ± 7 %, *n* = 10; *p* < 0.05 for USP2-69 at 0 mV; and 74 ± 8 % , *n* = 19; *p* < 0.05 for USP2-45 at 0 mV). In contrast, USP15, a related de-ubiquitylase [29], had no effect (Fig. 1a, b), suggesting that Ca_v_ channels are selectively regulated by USP2 de-ubiquitylases. The decrease in current amplitude was not caused by a shift in the current-voltage relationship (Fig. 1b, d) or inactivation properties of the channels (not shown). In particular, the midpoint of voltage-dependent activation (*V*_50,act_), calculated by fitting the current-voltage relationship as indicated in ‘Methods’, was not significatively altered by USP2-69 (−7 ± 3 mV versus −10 ± 1 mV in control; non-significant; NS) or USP2-45 (−9 ± 4 mV versus −10 ± 1 mV in control; NS), hence the amplitude of the effect of either USP2 splice variant can be directly compared at the same voltage. Interestingly, both USP2-69 and USP2-45 significantly increased the slope factor value (*k* = −9.1 ± 0.9 in USP2-69-transfected cells versus *k* = −5.9 ± 0.3 in control; *P* < 0.01; and *k* = −9.0 ± 1.1 in USP2-45-transfected cells versus *k* =−5.9 ± 0.4 in control; *P* < 0.05) which suggests that de-ubiquitylation of the channels may decrease their voltage-sensitivity. Figure 1c, d shows that the catalytically inactive mutant USP2-45C67A failed to regulate Ca_v_1.2 channels. Current densities recorded in USP2-45C67A-transfected cells were similar to control (control 38 ± 5 pA/pF at 0 mV, *n* = 16; and USP2C67A 37 ± 12 pA/pF at 0 mV, *n* = 11; NS), confirming that the effect of USP2-45 is mediated by its de-ubiquitylating activity. Further experiments were conducted with the USP2-45 isoform only.

### USP2-45 decreases the availability of Ca_v_1.2 channels at the plasma membrane

**Fig. 2:**
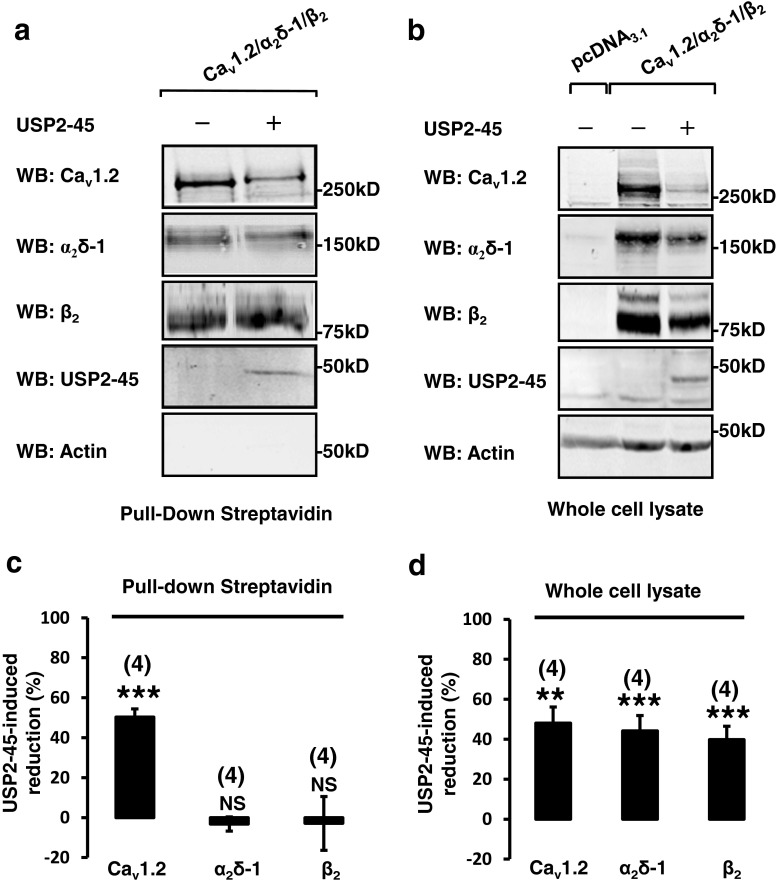
USP2-45 reduces the expression Ca_v_1.2 channels at the plasma membrane. **a**, **b** Surface biotinylation assays were performed in control and USP2-45-transfected HEK-293 cells. Steptavidin-covered beads were used to pull down the biotinylated proteins and their binding partners. Western blots (*WB*) show Ca_v_ channels and USP2-45 detected in the whole-cell lysates (**a**) and recovered by pull down with streptavidin (**b**) from a same experiment (*n* = 4). Note that the cytosolic USP2-45 was co-precipitated with the biotinylated fraction in (**a**), suggesting that this de-ubiquitylase binds to proteins expressed at the plasma membrane. As expected, the cytosolic β2 was co-purified with the biotinylated channels, because of their known interaction with Ca_v_1.2. As a control, another cytosolic protein (actin) was not recovered in the biotinylated fraction, indicating that the membrane integrity of the cells was preserved in our experimental conditions. **c** *Bar graphs* showing how USP2-45 alters the amount of Ca_v_ subunits recovered in pull-down experiments. The effect of USP2-45 was expressed as a percentage of reduction in the intensity of Ca_v_ protein bands relative to control. USP2-45 specifically decreased the membrane insertion of Ca_v_1.2, whereas the amount of biotinylated α_2_δ-1 and the proportion of the cytosolic β_2_ that co-purified with the biotinylated Ca_v_1.2 channels were unchanged. The number of independent experiments is indicated in *parentheses. NS* non-significant. ***p < 0.001 when compared with control. **d** *Bar graphs* showing the USP2-45-induced decrease in the amount of total Ca_v_ proteins (both biotinylated and non-biotinylated) recovered in the corresponding whole-cell lysates of cells exposed to biotin. The effect of USP2-45 was expressed as a percentage of reduction in the intensity of Ca_v_ protein bands relative to control. The decreased amount of Ca_v_ proteins detected in whole-cell lysates indicates that USP2-45 downregulated all three Ca_v_1.2, β_2_ and α_2_δ-1 subunits. **p < 0.01 and ***p < 0.001 when compared with control

A reduction of Ca_v_ current density may reflect a decrease in the number of channels trafficked to the plasma membrane. Hence, we examined the effect of USP2-45 on the plasma membrane expression of Ca_v_ channels by performing surface biotinylation assays. Figure 2a, c shows that USP2-45 reduced the amount of biotinylated Ca_v_1.2, whereas the surface abundance of α_2_δ-1 and co-precipitated β_2_ subunits were not affected. Remarkably, Western blots performed on the corresponding whole-cell lysates showed that USP2-45 decreased the protein amount of all three Ca_v_ subunits (Fig. 2b, d). It is noteworthy that the cytosolic β_2_ is pulled down together with the biotinylated Ca_v_1.2 (Fig. 2a). Interestingly, the amount of coprecipitated β suggested that the proportion of β associated with Ca_v_1.2 may be increased in USP2-45-transfected cells (by 120 ± 32 %, *n* = 4; *p* < 0.05 when calculating the ratio of co-precipitated β divided by the amount of biotinylated Ca_v_1.2). The same comparison cannot be made regarding α_2_δ-1, which is known to traffic to the cell surface independently of Ca_v_1.2 [18, 33]. Overall, these experiments showed that USP2-45 decreases the amount of Ca_v_1.2 channels inserted at the plasma membrane and that this downregulation is correlated with a reduction of all three (pore-forming and auxiliary) Ca_v_ proteins.

### USP2-45 promotes the de-ubiquitylation of both Ca_v_1.2 and α_2_δ-1 subunits

**Fig. 3:**
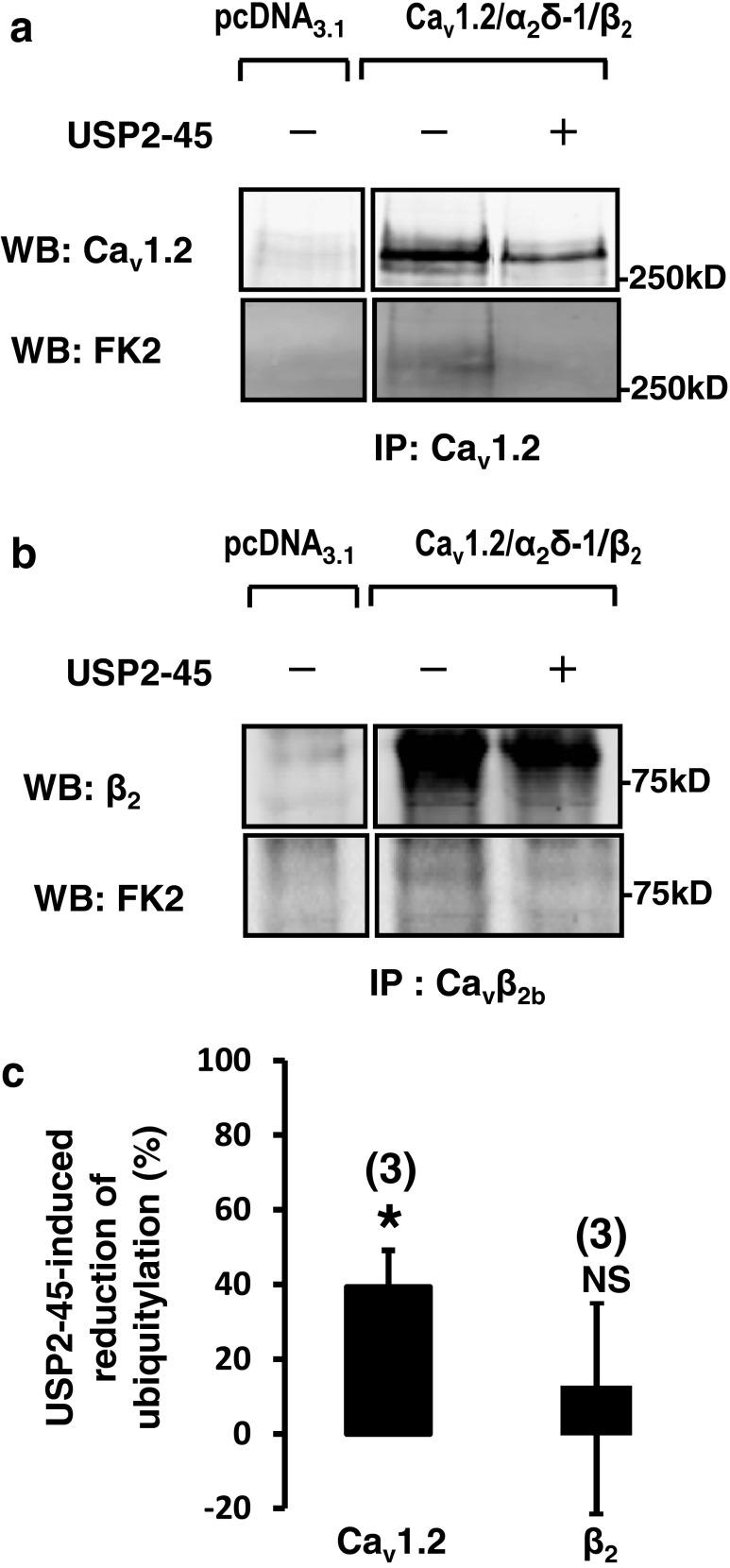
De-ubiquitylation of immunoprecipitated Ca_v_1.2 by USP2-45. **a** Immunoprecipitation of Ca_v_1.2 and **b** β_2_ subunits. Western blots were performed to confirm the purification of Ca_v_ subunits from HEK-293 cells, whilst anti-ubiquitin FK2 antibodies were used to detect the ubiquitylated channels. Control experiments show that Ca_v_1.2 and β_2_ are tonically ubiquitylated. **c** *Bar graph* showing that USP2-45 decreased the ubiquitylation level of Ca_v_1.2, but not β_2_ subunits. The intensity of the FK2 signal was normalized to take in account the reduction Ca_v_ protein (i.e. dividing by the intensity of the signal obtained with the corresponding Ca_v_ antibody), and the effect of USP2-45 was expressed as a percentage of change from the control value

**Fig. 4:**
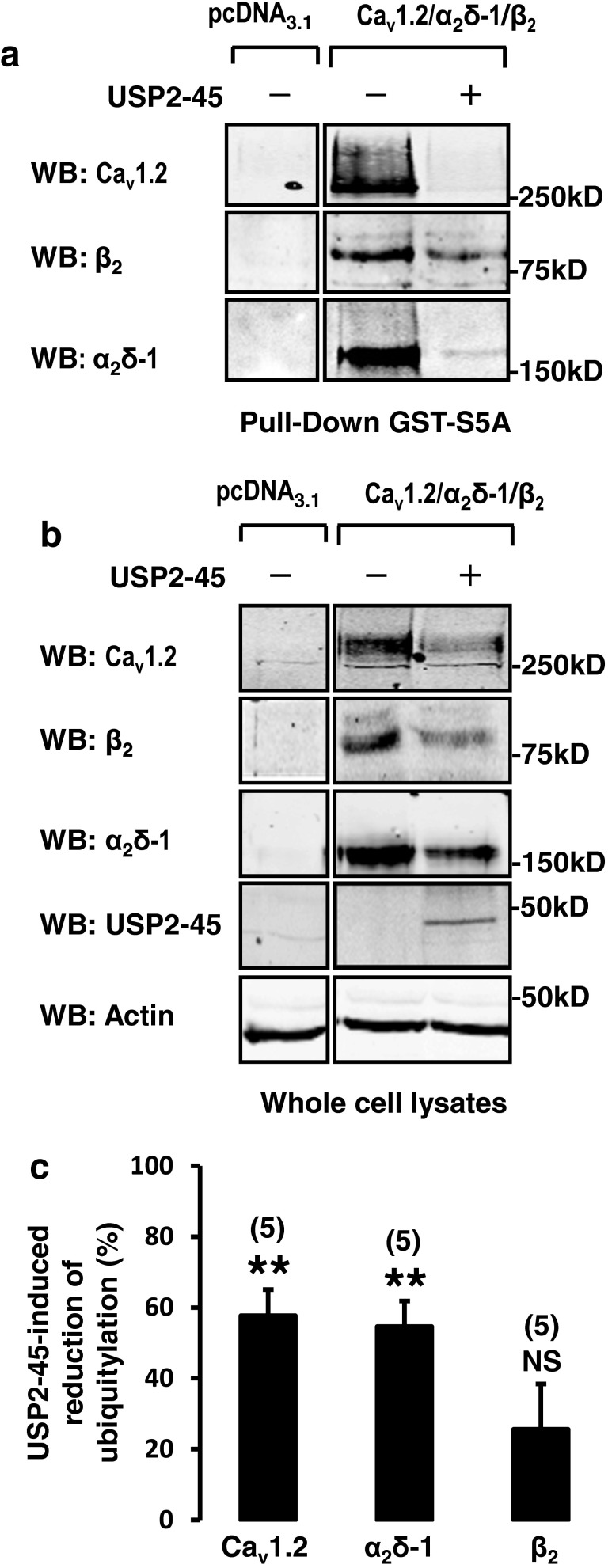
USP2-45-induced de-ubiquitylation of Ca_v_1.2 and α_2_δ-1 subunits. **a** Western blots showing the reduction of total Ca_v_1.2, β_2_ and Ca_v_α_2_δ-1 proteins in HEK-293 whole-cell lysates used for **b** pull down of ubiquitylated channels using ubiquitin binding GST-S5A (*n* = 5). Note that USP2-45 appears to reduce further the amount of Ca_v_1.2 and α_2_δ-1 subunits recovered in the pull-down assay. **c** *Bar graph* comparing the effect of USP2-45 on the ubiquitylation of the three different Ca_v_ subunits. To illustrate that the decrease in ubiquitylation of Ca_v_1.2 and α_2_δ-1 cannot be solely explained by the concomitant reduction in total Ca_v_ proteins, the intensity of each protein bands recovered by pull-down assays (shown in **b**) was divided by the intensity of the corresponding band in whole-cell lysates (ubiquitylated and non-ubiquitylated proteins shown in **a**). The data was expressed as a percentage change of this ratio relative to control. USP2-45 significantly decreased the ubiquitylation of Ca_v_1.2 and α_2_δ-1 but not β_2_ subunits. The number of experiments is indicated in *parentheses. NS* non-significant. ***p < 0.001 when compared with control

Next, we examined the effect of USP2-45 on Ca_v_ ubiquitylation. We immunoprecipitated Ca_v_1.2 and detected its level of ubiquitylation by using an FK2 anti-ubiquitin antibody, which recognizes both mono- and poly-ubiquitinated proteins. We found that USP2-45 reduced the ubiquitylation status of Ca_v_1.2 (Fig. 3a). Figure 3c shows a significant reduction in the ratio of intensity of the signal detected with the FK2 anti-ubiquitin antibody relative to the total amount of immunoprecipitated Ca_v_1.2, suggesting that USP2-45 de-ubiquitylates Ca_v_1.2 subunits. By contrast, USP2-45 did not significantly alter the ubiquitylation status of immunoprecipitated β_2_ subunits (Fig. 3c). Because of the lack of sensitivity of the α_2_δ-1 antibody, together with the downregulating effect of USP2-45, the amount of immunoprecipitated subunits was low and detection of ubiquitin on α_2_δ-1 was too weak to be reliably analysed using the FK2 antibody (not shown). Hence, we used an alternative approach and performed pull-down experiments using GST-S5a fusion proteins, which recognize poly-ubiquitylated proteins [12]. The specificity of the GST-S5a was demonstrated in our previous works [36]. In cells transfected with the channels only, GST-S5A pulled down all three Ca_v_ subunits, whereas no signal was recovered in cells transfected with the empty vector pcDNA3.1 only (Fig. 4a). This result is in line with the fact that the three subunits are tonically ubiquitylated in basal conditions [25, 36]. Most importantly, Fig. 4a shows that the amount of Ca_v_1.2 and α_2_δ-1 subunits recovered with GST-S5a was drastically reduced in cells co-transfected with USP2-45 compared to control cells transfected with the channels only. As expected from the experiments shown in Fig. 2b, d, USP2-45 also decreased the amount of proteins recovered in the corresponding whole-cell lysates (Fig. 4b, d). To correct for the reduction of Ca_v_ proteins and determine the relative change in ubiquitylation of each subunit, we calculated the ratio of ubiquitylated versus total (ubiquitylated and non-ubiquitylated) proteins. The effect of USP2-45 was expressed as a percentage of change from the control value (Fig. 4c). USP2-45 significantly decreased the ubiquitylation of Ca_v_1.2 and α_2_δ-1 but not β_2_ subunits. Altogether, these results indicate that both Ca_v_1.2 and α_2_δ-1, but not β_2_, are regulated by the USP2-45 de-ubiquitylase.

### The α_2_δ-1 subunit is essential for the regulation of Ca_v_ channels by USP-45

**Fig. 5:**
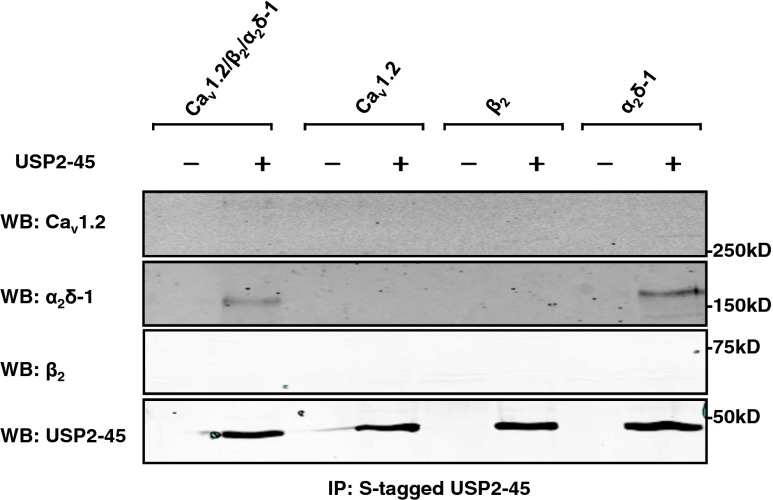
USP2-45 binds to α_2_δ-1 subunits. Pull-down experiments were performed on HEK-293 cells using biotinylated S-protein which recognizes an S-tag epitope inserted into USP2-45. Western blots show that USP2-45 co-precipitated with α_2_δ-1 but not Ca_v_1.2 nor β_2_ subunits (*n* = 3)

**Fig. 6:**
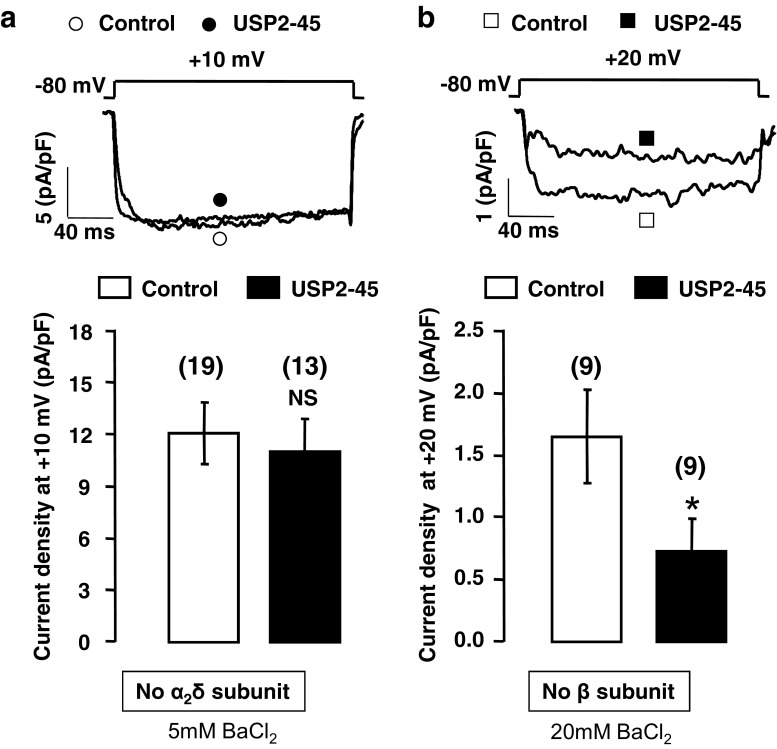
α_2_δ-1 is necessary for the USP2-45-induced decrease of Ca_v_1.2 currents. **a**, **b** Representative whole-cell current traces and corresponding bar graphs showing that USP2-45 failed to regulate Ca_v_1.2 channels in the absence of α_2_δ-1 subunits (**a**), whereas USP2-45 still decreases Ca_v_1.2 currents in the absence of β_2_ (**b**). tsA-201 cells were transfected with Ca_v_1.2/β_2_ alone (*white circle*, (control)) or together with USP2-45 (*black circle*) in **a** and Ca_v_1.2/α_2_δ-1 alone (*white square* (control)) or together with USP2-45 (*black square*) in **b**. As expected because of their known involvement in Ca_v_ trafficking, the absence of either subunit decreased Ca_v_ current densities (compared to Fig. 1). The lack of β subunit in particular dramatically decreased Ca_v_ currents, despite the charge carrier being increased to 20 mM BaCl_2_ in order to reliably quantify the decrease caused by USP2-45 on Ca_v_1.2/α_2_δ-1 channels. The data show the amplitude of the current densities recorded at 20 mV which generates the maximal current in the absence of β. As for prior experiments, 5 mM BaCl_2_ was used to record Ca_v_1.2/β_2_ currents. Currents densities are compared at 10 mV which generates the maximal current in the absence of α_2_δ-1. The number of cells is indicated in *parentheses. NS* non-significant. *p < 0.05 when compared with respective control

In support of a direct effect of USP2 on the channels, we found that USP2-45 co-immunoprecipitated with α_2_δ-1 but not with Ca_v_1.2 subunits (Fig. 5). As expected, we found no evidence for interaction of USP2-45 with β_2_ subunits (Fig. 5) which are not targeted by this de-ubiquitylase (Fig. 4a, c). These results suggest that USP2-45 binds only to the auxiliary subunit α_2_δ-1 and that this interaction is sufficient to promote the de-ubiquitylation of both α_2_δ-1 and Ca_v_1.2. To confirm the involvement of α_2_δ-1, we assessed the effect of USP2-45 on channels expressed without α_2_δ-1 or β subunits. We show that, in the absence of α_2_δ-1, USP2-45 did not alter Ca_v_1.2/β_2_ current densities (reduced by 9 ± 16 % at +10 mV, *n* = 19; NS; Fig. 6a), nor the current-voltage relationship: *V*_50,act_ for Ca_v_1.2/β_2_ channels was not significantly different between control (0.8 ± 1.0 mV, *n* = 11) and USP2-45-transfected cells (−2.6 ± 0.9 mV, *n* = 9; NS). By contrast, USP2-45 still reduced Ca_v_1.2/α_2_δ-1 current densities in the absence of β (by 56 ± 19 % at +20 mV, *n* = 9; *p* < 0.05; Fig. 6b), again without altering the current-voltage relationship (*V*_50,act_ = 6.8 ± 1.6 mV, *n* = 6 in control, versus *V*_50,act_ = 7.7 ± 4.6 mV, *n* = 3; NS; in USP2-45-transfected cells), confirming that, unlike α_2_δ-1, β subunits are not required for regulation of Ca_v_ channels by USP2-45. Altogether, these data demonstrate the paramount role of α_2_δ-1 in USP2-45-induced downregulation of Ca_v_ channels.

## Discussion

In this study, we identify the de-ubiquitylase USP2-45 as a novel regulator of Ca_v_ channels. We show that USP2-45 binds to α_2_δ-1, but not Ca_v_1.2 subunits, and that α_2_δ-1 is required for USP2-45-induced Ca_v_1.2 downregulation. This result suggests that the first critical event is the binding of USP2-45 to the auxiliary α_2_δ-1 subunit which may act as an anchor allowing for USP2-45 to de-ubiquitylate Ca_v_1.2 channels. USP2-45 promotes the de-ubiquitylation of both Ca_v_1.2 and α_2_δ-1 subunits, reducing the availability of Ca_v_1.2 channels at the plasma membrane. This result is intriguing because de-ubiquitylation is most often associated with stabilization of proteins at the cell surface [15, 38]. Nonetheless, USP2-45 was also reported to induce the degradation of a mineralocorticoid receptor, by disrupting its association with a stabilizing partner which preferentially interacts with the ubiquitylated form of the receptor [16]. Overexpression of USP2 was also shown to reduce p53 stability [39], and another de-ubiquitylase USP8 (also called UBPY) promotes epithelial growth factor receptor degradation [4, 32, 37]. De-ubiquitylation may also serve to allow the recycling of ubiquitin moieties attached to the proteins prior to degradation, as reported for epithelial growth factor receptors [3, 37]. In addition, the binding of USP2-45 to α_2_δ-1 may disrupt the chaperone role of this subunit towards Ca_v_1.2, leading to the reduction of Ca_v_1.2 surface expression. The conserved amount of membrane-associated α_2_δ-1 in USP2-45-transfected cells, revealed by surface biotinylation assays, could be explained by the fact that these subunits are expressed at the cell surface as both single units and complexed with the main pore-forming Ca_v_ α1 subunits [18, 33]. It is expected that the remaining fraction of α_2_δ-1 subunits, which have not yet been sent for degradation, continues to be efficiently targeted alone to the plasma membrane, independently of Ca_v_1.2. Noteworthingly, α_2_δ-1 is described as both transmembrane proteins [34] and extracellular glycosylphosphatidylinositol (GPI)-anchored proteins [11] and may coexist in the two forms [11]. Because USP2-45 specifically binds to α_2_δ-1, one can assume that the cytosolic USP2-45 recovered in surface biotinylation assays is co-precipitated with the biotinylated α_2_δ-1 subunits. Importantly, this result suggests that at least a fraction of α_2_δ-1 is a transmembrane protein, accessible for and able to retain the binding of the cytosolic USP2-45. Remarkably, our study identifies USP2-45 as a novel binding partner for α_2_δ-1. To date, apart from α1 (the pore-forming subunit itself), the only other known α_2_δ-1-interacting proteins were components of the extracellular matrix called thrombospondins (TSP1, TSP2 and TSP4) [14, 21] and a subunit of a mitochondrial ATP synthase complex (ATP5b) [19]. The α_2_δ-1 subunit also binds to the anti-allodynic drug gabapentin used for the treatment of neuropathic pain [20].

One intriguing finding of our study is that although the Ca_v_ subunit β is not itself de-ubiquitylated by USP2-45, the total amount of β available in USP2-45-transfected cells is reduced together with Ca_v_1.2 and α_2_δ-1. One possible explanation is that USP2-45 preferentially regulates Ca_v_1.2 when associated with both accessory subunits β and α_2_δ-1, which may then be concomitantly sent for degradation. Alternatively, an excess of free β may be degraded consequently to USP2-45-induced degradation of Ca_v_1.2. In favour of this hypothesis, a recent report showed that β subunits which fail to associate with Ca_v_2.2 are degraded by the proteasome [42]. Importantly, both β and α_2_δ-1 can independently promote Ca_v_1.2 insertion [9, 17], but only α_2_δ-1-associated channels are sensitive to USP2-45. Hence, the fraction of Ca_v_ channels insensitive to USP2-45 and able to reach the plasma membrane would be mostly composed of Ca_v_1.2/β channels. In favour of this model, we found that the fraction of Ca_v_ channels expressed at the plasma membrane of USP2-45-transfected cells is slightly enriched in Ca_v_β.

In our study, we exogeneously expressed cloned cardiac Ca_v_ channels and USP in mammalian cell lines but the extent of such regulation and the role of α_2_δ-1 in the de-ubiquitylation of Ca_v_ channels in cardiac myocytes remain to be investigated. The α_2_δ-1 subunits are also associated with Ca_v_2 channels which are predominantly expressed in neurons [8]. α_2_δ-1 was reported to control synaptic release probability by regulating presynaptic Ca_v_2 channel availability [23]. Ca_v_2 is also ubiquitylated [2]. Hence, it would be worth assessing whether α_2_δ-1 enables USP2-45, which is known to be expressed in the brain [22], to also de-ubiquitylate neuronal Ca_v_2 channels.

Overall, our results argue for a dual role of α_2_δ_1_ on Ca_v_ trafficking: increasing (as previously shown [16, 7, 17]) or decreasing Ca_v_ expression at the plasma membrane. We propose that USP2-45 acts as a switch to redirect the action of α_2_δ_1_ towards a decrease in Ca_v_ channels availability, thus revealing a new role both for α_2_δ_1_ and USP2-45 in calcium signalling.

## Abbreviations

USPs: Ubiquitin-specific proteases
Ca_v_: Voltage-gated calcium channel
